# Regular pulse checks for patients with non-cardioembolic stroke in rehabilitation hospitals to improve recognition and detection of atrial fibrillation (the ESCORT study): protocol for a prospective multicenter observational study

**DOI:** 10.3389/fneur.2023.1247020

**Published:** 2023-08-16

**Authors:** Takehiro Katano, Satoshi Suda, Tomohiro Ohta, Mitsusuke Miyagami, Yuzo Kodaira, Chimori Konaka, Masakazu Nagashima, Kazumi Kimura

**Affiliations:** ^1^Department of Neurology, Nippon Medical School Hospital, Tokyo, Japan; ^2^Department of Stroke Neurology, Saitama Medical University International Medical Center, Saitama, Japan; ^3^Department of Internal Medicine, Araki Kinen Tokyo River Side Hospital, Tokyo, Japan; ^4^Department of Rehabilitation and Neurosurgery, Takenozuka Nohsinkei Rehabilitation Hospital, Tokyo, Japan; ^5^Department of Surgery, Flowers and Forest Tokyo Hospital, Tokyo, Japan; ^6^Department of Rehabilitation, Akabane Rehabilitation Hospital, Tokyo, Japan; ^7^Department of Orthopedics, Katsushika Rehabilitation Hospital, Tokyo, Japan

**Keywords:** cryptogenic stroke, paroxysmal atrial fibrillation, protocol, rehabilitation, pulse check

## Abstract

**Background:**

Cryptogenic stroke (CS) are heterogeneous in origin; however, most CS are embolic mechanism. Paroxysmal atrial fibrillation (AF) is suspected to be a major type of CS that leads to severe cerebral infarction without anticoagulant use. Therefore, the identification of AF is vital in patients with CS. However, patients are often unaware of AF because they have no symptoms, and AF may not be detected on an electrocardiogram (ECG) or Holter ECG on admission. After patients with stroke are treated in the acute phase, they are promptly transferred to a rehabilitation hospital for functional recovery. Once the patient is transferred to a hospital, a few attempts are made to detect AF. In addition, rehabilitation therapists are considered to have insufficient awareness of the possibility of undiagnosed AF.

**Objective:**

This study aimed to increase the understanding of the importance of AF detection in patients with ischemic stroke among therapists in rehabilitation hospitals and to investigate whether regular pulse screening can aid in the detection of AF. If AF was detected, we determined the rate and timing of AF detection and identified the patient characteristics.

**Methods:**

This multicenter prospective observational study aimed to detect AF in patients with non-cardiac stroke at rehabilitation hospitals. Therapists performed pulse checks before, during, and after rehabilitation. If arrhythmia or tachycardia was detected, an ECG was performed, and the physician checked for AF. If the patient complained of chest symptoms, electrocardiography (ECG) was performed to check for AF. We investigated the characteristics, laboratory data, cognitive status, complications, such as stroke recurrence, and functional outcomes of patients with AF.

**Results:**

The study is in the enrollment phase. Recruitment began in September 2022 and will end in August 2023. Patients have provided written informed consent. The main results have been submitted for publication in your journal.

**Conclusion:**

The findings of this study will help identify patients with AF in rehabilitation hospitals and improve awareness among therapists.

## 1. Introduction

Approximately 15%–40% of all ischemic strokes are characterized as cryptogenic stroke (CS) (1). CS are heterogeneous in origin; however, many CS are thought to exhibit embolic mechanisms. Paroxysmal atrial fibrillation (AF) is suspected to be the major cause of CS. AF attributable to large- or small-vessel disease has also been reported in stroke patients (2).

The number of patients with AF increases with age (3, 4); however, AF is sometimes difficult to detect. AF is classified as persistent AF if it lasts for more than 1 week or as paroxysmal AF if it disappears within 1 week. Paroxysmal AF is sometimes unrecognized by patients because it is asymptomatic (5) and may not be detected on electrocardiography (ECG) or Holter ECG examination on admission.

Paroxysmal and persistent AF are associated with a high risk of recurrence without anticoagulant therapy (6); therefore, we treated the patients with anticoagulant therapy to prevent stroke due to AF. Current Japanese guidelines for stroke recommend that stroke in which AF is not detected should be classified as non-cardiogenic stroke and treated with antiplatelet agents (aspirin, clopidogrel, prasgrel, and cilostazol) to prevent recurrence. However, when atrial fibrillation is observed, antiplatelet drugs are insufficient to prevent stroke recurrence, and anticoagulants (warfarin, dabigatran, rivaroxaban, apixaban, edoxaban) are more effective in preventing recurrence (7, 8). Therefore, it is important to actively identify patients with stroke without AF.

Recently, stroke care has changed considerably with advances in acute therapy and shortened length of hospitalization. Once patients are transferred to a rehabilitation hospital, fewer attempts are made to detect AF. In addition, rehabilitation therapists have insufficient awareness of the possibility of undiagnosed AF and the importance of AF detection. In this study, we aimed to increase the understanding of the importance of AF detection among therapists in rehabilitation hospitals and investigate whether regular pulse screening can aid in the detection of AF. If the feasibility and usefulness of regular pulse testing during rehabilitation are confirmed, the detection of AF after stroke can be recommended.

This study also aimed to clarify the rate and timing of AF detection by performing pulse testing.

## 2. Methods

### 2.1. Study design

The regular pulse check for patients with non-cardioembolic stroke in a rehabilitation hospital to improve recognition and detection of atrial fibrillation (ESCORT) study is an observational, multicenter, prospective registry of patients diagnosed with ischemic stroke.

### 2.2. Ethics approval

Ethical approval for this study was obtained from the ethics committee of Nippon Medical School (A-2021-067) and the relevant ethics committees of all participating centers.

Written informed consent was obtained from all the patients or their family members before participating in the study. This study was registered with University Hospital Medical Information.

Network (UMIN) Clinical Trial Registry (UMIN000048536).

### 2.3. Patient population

Patient enrollment for this ongoing study began in September 2022 at six medical partner institutions in Tokyo, Japan. The inclusion criteria were as follows: (1) patients diagnosed with non-cardiogenic stroke, (2) patients aged >20 years, and (3) patients or their families who agreed to participate in the study and provided informed consent. Patients with insertable cardiac monitor (ICM) were also participated if they met the inclusion criteria. The exclusion criteria were as follows: (1) patients who are currently participating or will participate in another intervention trial, (2) patients with AF or atrial flutter, (3) patients diagnosed with less than 1 year to live, and (4) patients judged by their doctor to be ineligible to participate in the study. A full description of the inclusion and exclusion criteria is presented in Table 1. Trained stroke rehabilitation nurses and physicians educated the registered patients on pulse palpation according to a standardized protocol that focuses on evaluating the heart rate and rhythm regularity. First, rehabilitation therapists were educated about the health implications of AF (i.e., the risk of ischemic stroke, thromboembolism, heart failure, and mortality), clinical presentation, and its frequent asymptomatic nature. The potential health benefits of the intervention were also explained.

**Table 1:** Inclusion and exclusion criteria.

**Inclusion criteria**
Patients who have been diagnosed with non-cardiogenic stroke
Patients aged over 20 years
Patients or their family agreed in writing
**Exclusion criteria**
Patient is currently participating or will participate in another intervention trial
Patient has AF or atrial flutter
Patient diagnosed with less than 1 year to live
Patients judged by their doctor to be ineligible to participate in the study

### 2.4. Evaluations and follow-up

**Figure 1:**
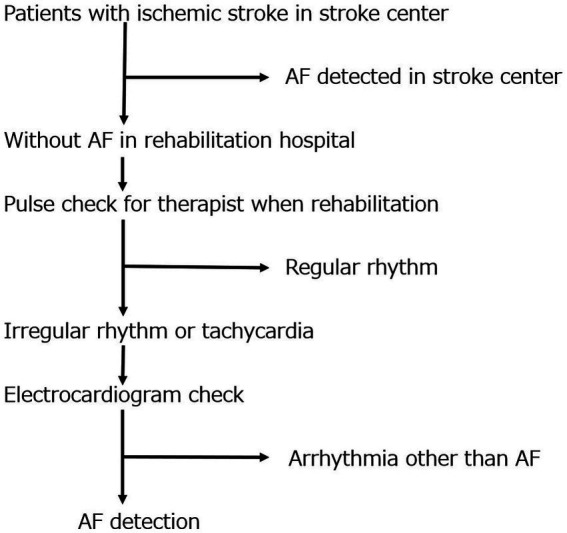
Study flowchart. This flowchart illustrates the design of this study. It shows the flow chart from stroke diagnosis at the stroke center to pulse and AF diagnosis at the rehabilitation hospital. AF, atrial fibrillation.

The following parameters were evaluated: (1) feasibility of pulse checks in rehabilitation hospitals, (2) proportion of AF detection, and (3) proportion of initiation of anticoagulant therapy. Patients usually undergo rehabilitation therapy 3 times daily at rehabilitation hospitals. Trained rehabilitation staff performed pulse checks for each patient before, during, and after rehabilitation (9 times daily). If an irregular pulse and tachycardia (heart rate of 120 beats/min or faster) were detected, a 12-lead ECG immediately (within 5 min) after pulse assessment was essential to confirm or rule out AF (Figure 1). For patients with identified AF, the prescription will be changed to an anticoagulant at the rehabilitation hospital and the study office will be contacted. If the attending physician at the rehabilitation hospital has concerns about the prescription, the study office will consult about the prescription. The duration of the regular pulse monitoring is all periods from obtaining consent to admission in rehabilitation hospital.

### 2.5. Data collection

The following study data will be encrypted and stored on a website: patient characteristics, electrocardiogram data, laboratory data, hospital events, and discharge status (Table 2). The data will be accessible only to study researchers with appropriate training and ethics review board approval, and the study steering committee will independently monitor the data.

**Table 2:** Detailed survey in this study.

**Patient characteristics**
Age, sex, height, weight, modified Rankin Scale (mRS) score, type of stroke, hypertension, dyslipidemia, diabetes mellitus, chronic kidney disease, congestive heart failure, cerebrovascular disease, peripheral artery disease, peripheral artery disease, thromboembolism, active cancer, dementia, alcohol intake, smoking, antithrombotic therapy, functional independence measure (FIM), and mini mental state examination stroke (MMSE)
**Electrocardiogram**
PR interval, QTc interval, and supraventricular extrasystole
**Blood examination**
Routine blood biochemistry examinations, including creatinine clearance, blood glucose, low-density lipoprotein cholesterol and measurements of D-dimers, brain natriuretic peptide (BNP), and N-terminal prohormones of the BNP
**Events in the hospital**
Infection (urinary tract infection, pneumonia, cholecystitis, and other), recurrent stroke, falls
**Status at discharge**
mRS at discharge, MMSE at discharge, FIM at discharge, and discharge destination (home, hospital, convalescent home, other)

### 2.6. Sample size

Notably, this study has not been previously reported. The number of possible participants in the study in a year was calculated from the number of admissions at participating facilities in the previous year and set at 1000.

### 2.7. Data analysis plan

We will examine the proportion of patients with AF detected, timing of detection, and characteristics of patients with AF. Clinical characteristics such as sex, age, laboratory data, echocardiography data, and brain imaging data, and outcomes will be compared between the patients in the AF and non-AF group. Sub-group analyses will be planned for patients who used other long-term electrocardiographic monitors, such as ICM. All statistical analyses will be performed using the SPSS software (version 27; SPSS Japan, Inc., Tokyo, Japan). The results will be considered statistically significant at *p* < 0.05. Univariate analyses will be performed using *χ*^2^ and Mann–Whitney *U* tests, as appropriate. Data will be presented as medians and interquartile ranges. Categorical variables will be presented as frequencies and percentages.

### 2.8. Study organization

The ESCORT study was organized by a central coordinating center in the Department of Neurology, Nippon Medical School, and is being conducted at six medical partner institutions in Tokyo, Japan.

## 3. Results

Study recruitment began in September 2022 and will end in August 2023. The patients provided written informed consent and are currently in the enrollment phase. Data clean-up and analyses are projected to be completed by August 2023, and the results are expected to be submitted for peer-reviewed publications.

## 4. Discussion

AF after non-cardiac stroke is often asymptomatic and paroxysmal; thus, AF is unlikely to be detected by strategies based on symptom-driven monitoring. AF is typically detected in stroke patients after admission, but can be missed during the acute phase in stroke centers. Long-term ECG monitoring improves AF detection rates (8–12); however, using this method as an insertable cardiac monitor is invasive and expensive. In Japan, patients are transferred to rehabilitation hospitals after acute treatment, which makes continuous ECG monitoring difficult. The Japanese Arrhythmia Guidelines recommends pulse check, a simple and inexpensive method, as a Class 1 method for AF screening. Early detection of atrial fibrillation is important to prevent recurrence; however, not all cases are detected immediately after a stroke. Thus, a method that enables continuous arrhythmia assessment after the acute phase in rehabilitation hospitals is required. Pulse monitoring is a simple and inexpensive method. If this project confirms the feasibility and usefulness of pulse checks for detecting AF and starting anticoagulant therapy in rehabilitation hospitals, it will be especially useful for the prevention of cardioembolic stroke, heart failure, and mortality.

## 5. Conclusion

This ESCORT study aimed to increase the awareness of AF detection among therapists and its utility in rehabilitation hospitals. Additionally, we hope that the findings of this study will facilitate the identification of previously undetected cases of AF among stroke patients in rehabilitation hospitals, leading to improved prevention of recurrent strokes.

## Data availability statement

The raw data supporting the conclusions of this article will be made available by the authors, without undue reservation.

## Ethics statement

The studies involving humans were approved by the ethics committee of Nippon Medical School. The studies were conducted in accordance with the local legislation and institutional requirements. The participants provided their written informed consent to participate in this study.

## Author contributions

TK conceived and designed the study and drafted the manuscript. SS conceived and designed the study. TO, MM, YK, CK, and MN collected the data. KK critically revised the manuscript. All authors contributed to the article and approved the submitted version.

## Funding

This study received funding from Pfizer Co., Ltd. The funder was not involved in the study design, collection, analysis, interpretation of data, the writing of this article or the decision to submit it for publication.

## Conflict of interest

The authors declare that the research was conducted in the absence of any commercial or financial relationships that could be construed as a potential conflict of interest.

## Publisher’s note

All claims expressed in this article are solely those of the authors and do not necessarily represent those of their affiliated organizations, or those of the publisher, the editors and the reviewers. Any product that may be evaluated in this article, or claim that may be made by its manufacturer, is not guaranteed or endorsed by the publisher.
